# Crystal structure, Hirshfeld surface analysis, DFT and the mol­ecular docking studies of 3-(2-chloro­acet­yl)-2,4,6,8-tetra­phenyl-3,7-di­azabicyclo­[3.3.1]nonan-9-one

**DOI:** 10.1107/S2056989024008302

**Published:** 2024-08-30

**Authors:** Sivagnanam Divyabharathi, Anjalai Ramachandran Karthiga, Rajans Reshwen Shalo, Krishnan Rajeswari, Thankakan Vidhyasagar, Sivashanmugam Selvanayagam

**Affiliations:** a Department of Chemistry Annamalai University, Annamalainagar Chidambaram 608 002 India; b PG & Research Department of Chemistry, Government Arts College, Chidambaram 608 102, India; c PG & Research Department of Physics, Government Arts College, Melur 625 106, India; Vienna University of Technology, Austria

**Keywords:** crystal structure, aza­bicyclo derivatives, boat–boat conformation, C—H⋯π inter­molecular inter­actions, Hirshfeld surface analysis

## Abstract

In the title compound, C_33_H_29_ClN_2_O_2_, the two piperidine rings of the di­aza­bicyclo moiety adopt distorted-chair conformations. Inter­molecular C—H⋯π inter­actions are mainly responsible for the crystal packing. The inter­molecular inter­actions were qu­anti­fied and analysed using Hirshfeld surface analysis, revealing that H⋯H inter­actions contribute most to the crystal packing (52.3%). The mol­ecular structure was further optimized by density functional theory (DFT) at the B3LYP/6–31 G(d,p) level and is compared with the experimentally determined mol­ecular structure in the solid state.

## Abstract 2

In the bicyclic title compound, C_33_H_29_ClN_2_O_2_, the two piperidine rings of the di­aza­bicylco moiety adopt distorted-chair conformations.

## 1. Chemical context

The 3,7-di­aza­bicyclo­[3.3.1]nonane core structure is present in many naturally occurring lupin alkaloids such as lupanine, sparteine, isolupanine and hy­droxy­lupanine. The bridged bicyclic ring system present in 3-aza­bicyclo­[3.3.1]-9-ones [3-ABN] and 3,7-di­aza­bicyclo­[3.3.1]nonan-9-ones [3-DABN] can adopt twin chair, chair–boat or twin boat stereochemical conformations (Srikrishna & Vijaykumar, 1998 ▸; Pathak *et al.*, 2007 ▸; Vijayakumar & Sundaravadivelu, 2005 ▸). Syntheses and stereochemistries of these bicyclic compounds have extensively been studied by several groups (Jeyaraman & Avila, 1981 ▸), and their biological potencies have also been well established (Parthiban *et al.*, 2009 ▸). Inter­estingly, the *N*-nitroso derivatives of 3-DABN show distorted chair–chair conformations (Natarajan & Mathews, 2011 ▸), and 3,7-dialk­yl/diacyl-3,7-di­aza­bicyclo­nona­nes serve as stimulus-sensitive mol­ecular switches and lipid bilayer modifiers (Veremeeva *et al.*, 2014 ▸, 2019 ▸).
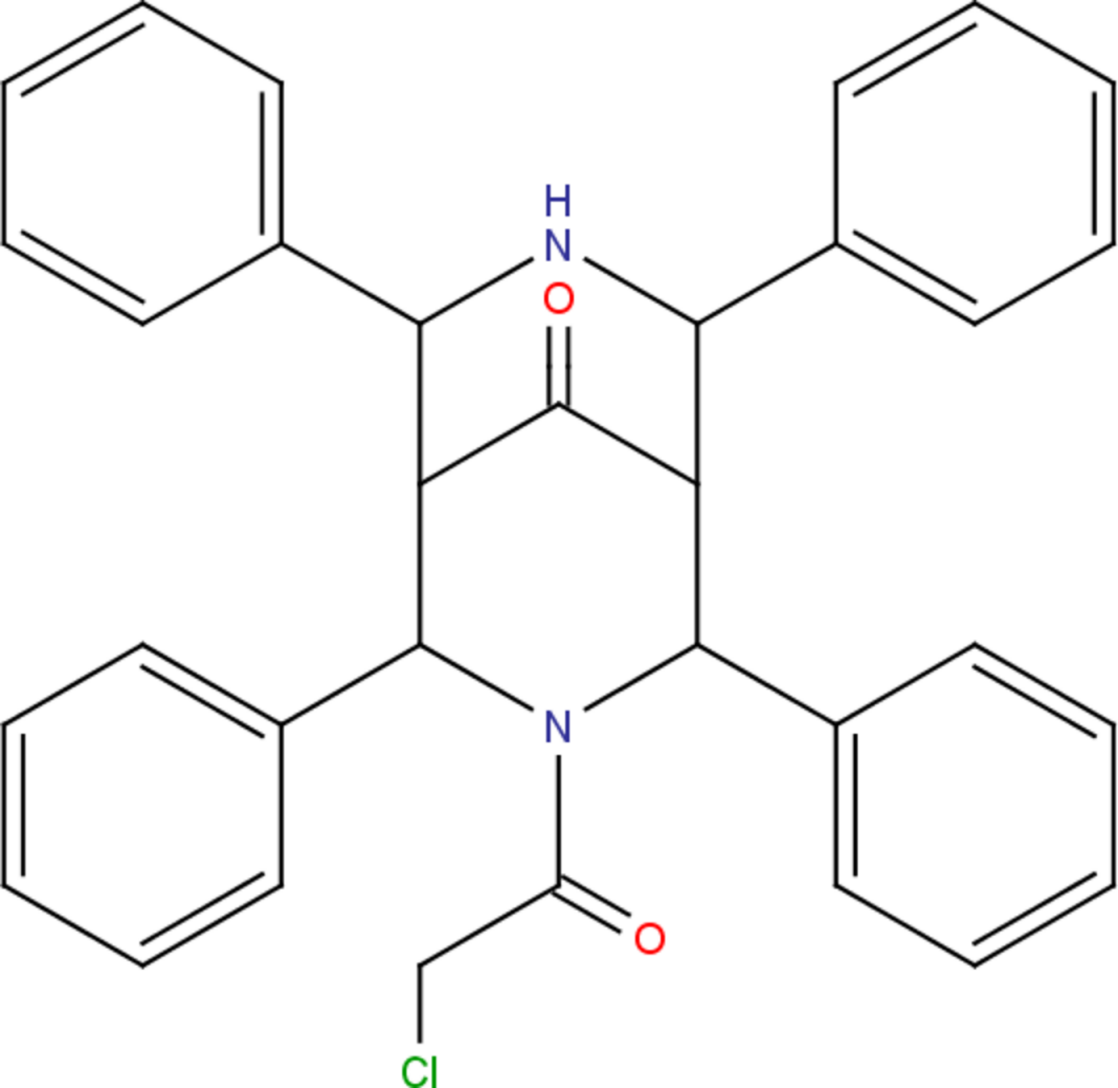


In the present work, the synthesis, structural and computational studies of 3-(2-chloro­acet­yl)-2,4,6,8-tetra­phen­yl-3,7-di­aza­bicyclo­[3.3.1]nonan-9-one, (I), a similar analogue of 3-DABN, is reported.

## 2. Structural commentary

**Figure 1:**
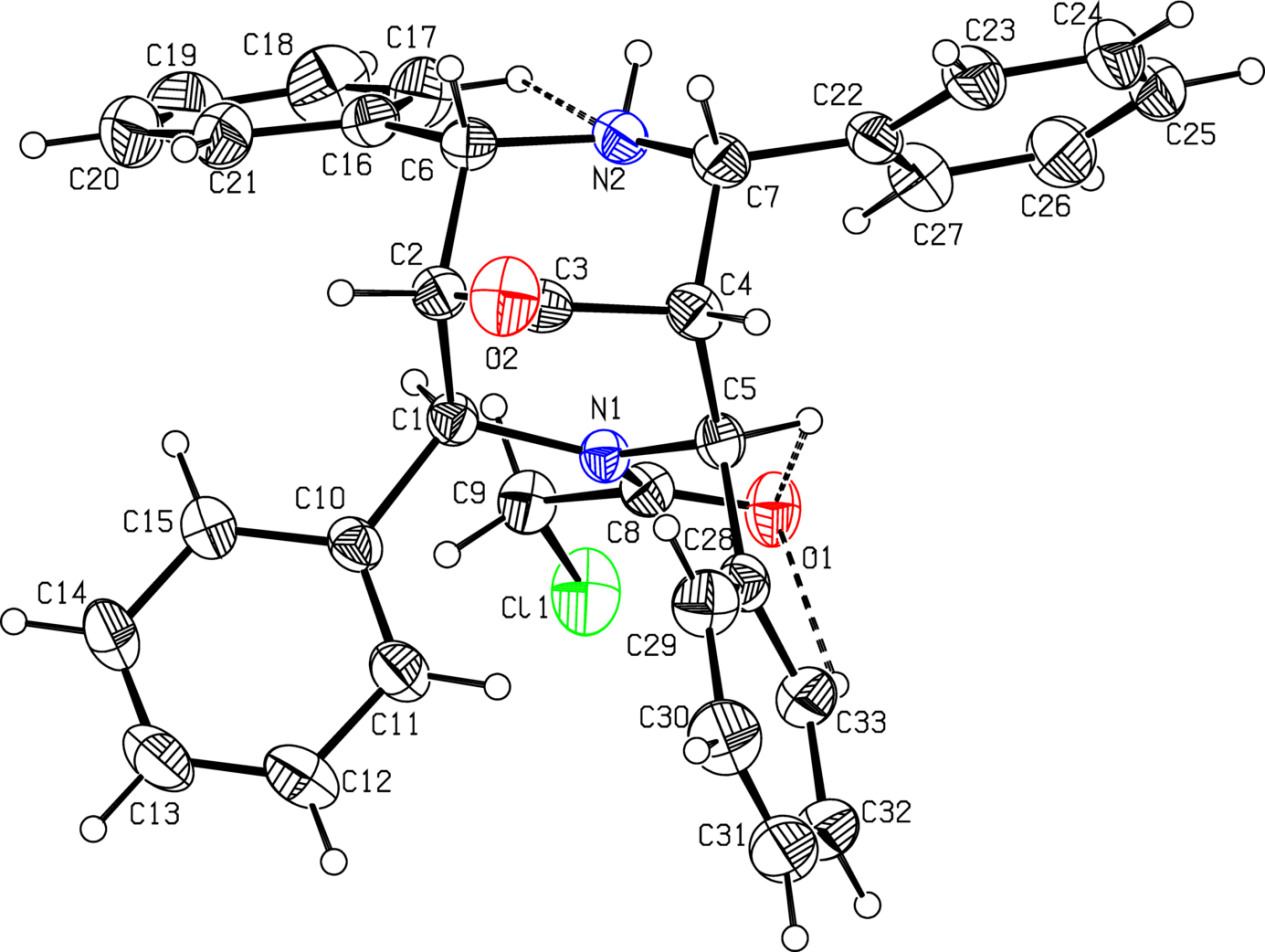
A view of the mol­ecular structure of (I), showing the atom labelling. Displacement ellipsoids are drawn at the 30% probability level. Intra­molecular hydrogen bonds are shown as dashed lines.

The mol­ecular structure of (I) is displayed in Fig. 1 ▸. The chloro­acetyl group (C8/O1/C9/Cl1) and the phenyl ring (C10–C15) are perpendicular with each other and make a dihedral angle of 89.8 (1)°. The chloro­acetyl group is planar with a maximum deviation of 0.080 (1) Å for atom C8.

The two piperidine rings (N1/C1–C5) and (N2/C6/C2–C4/C7) in the di­aza­bicyclo moiety adopt distorted chair conformations, with puckering parameters (Cremer & Pople, 1975 ▸) of *Q* = 0.487 (2) Å, θ = 157.5 (2)°, φ = 6.6 (6)° for ring (N1/C1–C5) and 0.628 (2) Å, θ = 5.8 (2)° and φ = 194.5 (16)° for ring (N2/C6/C2–C4/C7). Atoms C5 and C1 in the (N1/C1–C5) ring deviate by 0.489 (1) and −0.599 (2) Å, respectively, from the least-squares plane through the remaining four atoms. Similarly, atoms C4 and C6 in the (N2/C6/C2–C4/C7) ring deviate by 0.781 (1) and −0.695 (1) Å respectively, from the least-squares plane through the remaining four atoms. The eight-membered ring (N1/C1/C2/C6/N2/C7/C4/C5) of the aza­bicyclo moiety has a boat–boat conformation, with puckering parameters *q*_2_ = 1.565 (2) Å, *q*_3_ = *q*_4_ = 0.086 (2) Å and θ_2_ = 86.8 (2)° (Evans & Boeyens, 1988 ▸).

**Figure 2:**
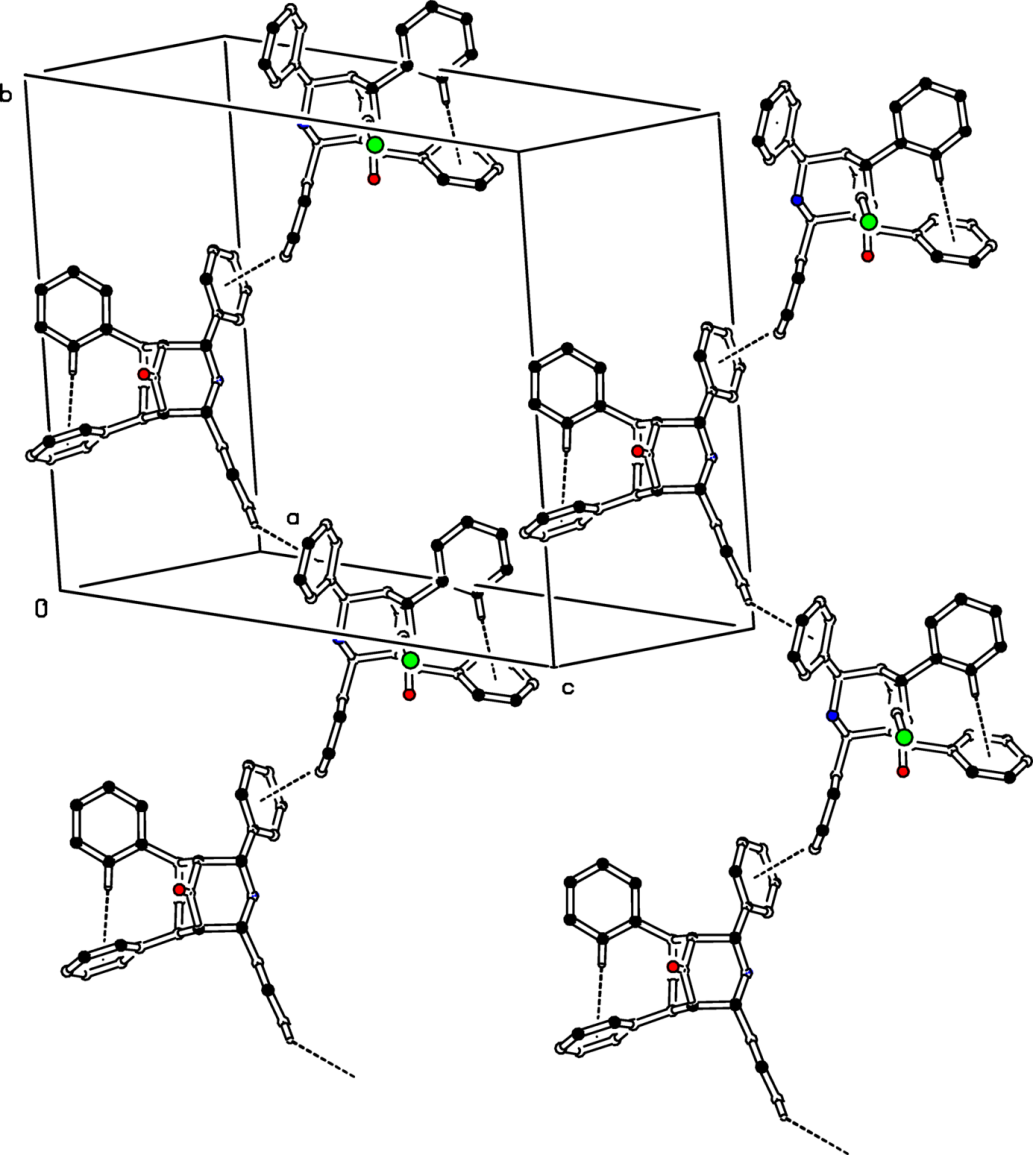
The crystal packing of (I). The intra- and inter­molecular C—H⋯π inter­actions are shown as dashed lines. For clarity, H atoms not involved in these inter­actions have been omitted.

Intra­molecular C17—H17⋯N2 and C5—H5⋯O1 contacts, forming two *S*(5) ring motifs (Bernstein *et al.*,1995 ▸), lead to the stabilization of the mol­ecular conformation, supplemented by the C33—H33⋯O1 contact, forming an *S*(6) ring motif (Fig. 1 ▸, Table 1 ▸). An intra­molecular C—H⋯π inter­action is observed (C11—H11⋯*Cg*1) involving the centroid of the C28–C33 benzene ring (Table 1 ▸, Fig. 2 ▸).

## 3. Supra­molecular features

In the crystal, mol­ecules are linked into a *C*(10) chain motif by C—H⋯π inter­actions, C24—H224⋯*Cg*2, where *Cg*2 is the centroid of the symmetry-related mol­ecule C16–C21 benzene ring at (1 − *x*, −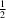 + *y*, 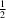 − *z*) (Table 1 ▸). This *C*(10) chain runs in a helical manner parallel to [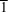
10] (Fig. 2 ▸). It is inter­esting to note that the amine function (N2—H2) is not involved in any inter­molecular inter­actions.

## 4. Hirshfeld surface analysis

**Figure 3:**
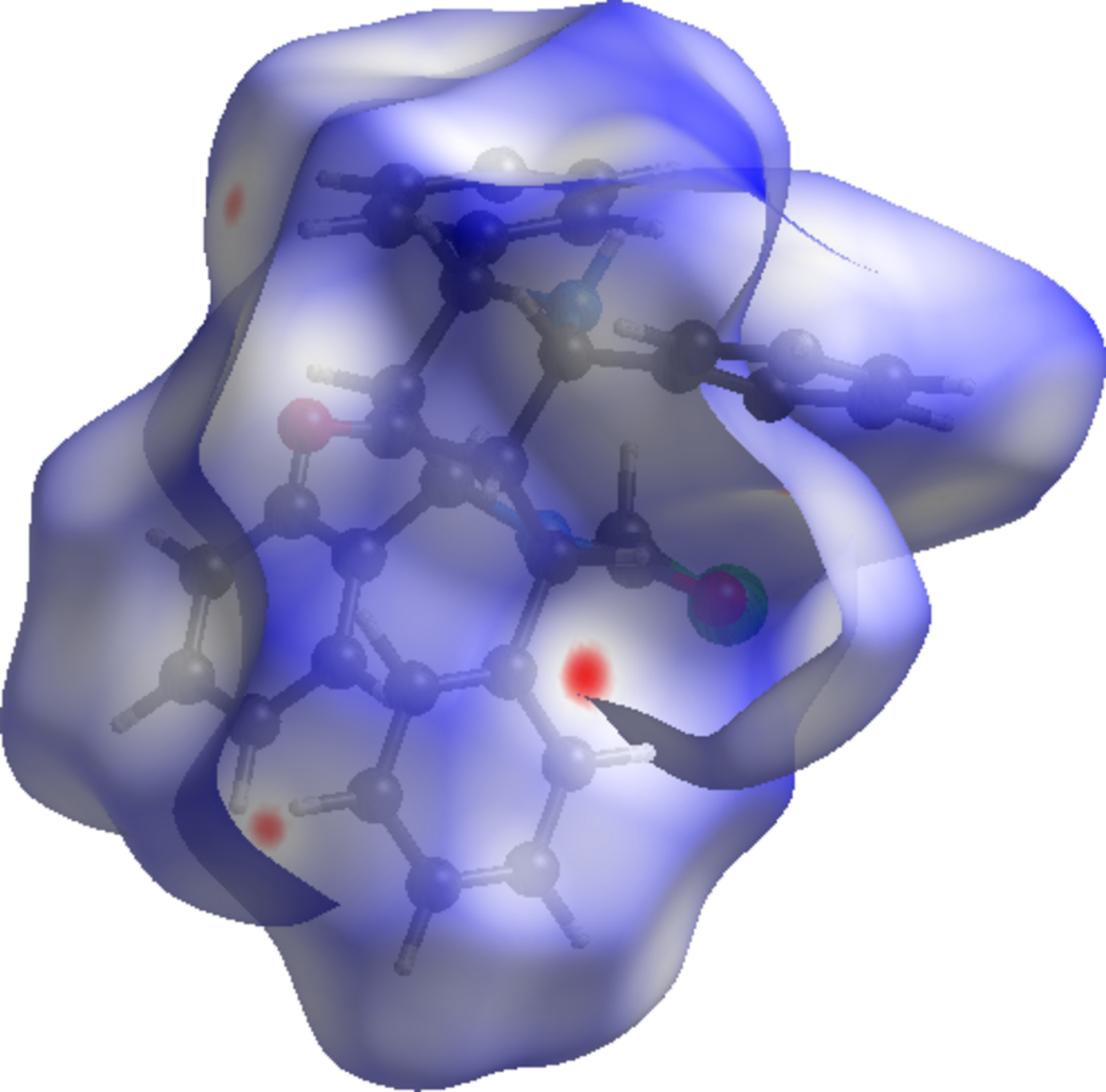
A view of the Hirshfeld surface mapped over *d*_norm_ for (I).

To characterize the inter­molecular inter­actions in (I), a Hirshfeld surface (HS) analysis (Spackman & Jayatilaka, 2009 ▸) was carried out using *CrystalExplorer 21* (Spackman *et al.*, 2021 ▸) and the associated two-dimensional fingerprint plots (McKinnon *et al.*, 2007 ▸) were generated. The HS mapped over *d*_norm_ in the range −0.0876 to +1.5105 a.u. is illustrated in Fig. 3 ▸, using colours to indicate contacts that are shorter (red areas), equal to (white areas), or longer than (blue areas) the sum of the van der Waals radii (Ashfaq *et al.*, 2021 ▸).

**Figure 4:**
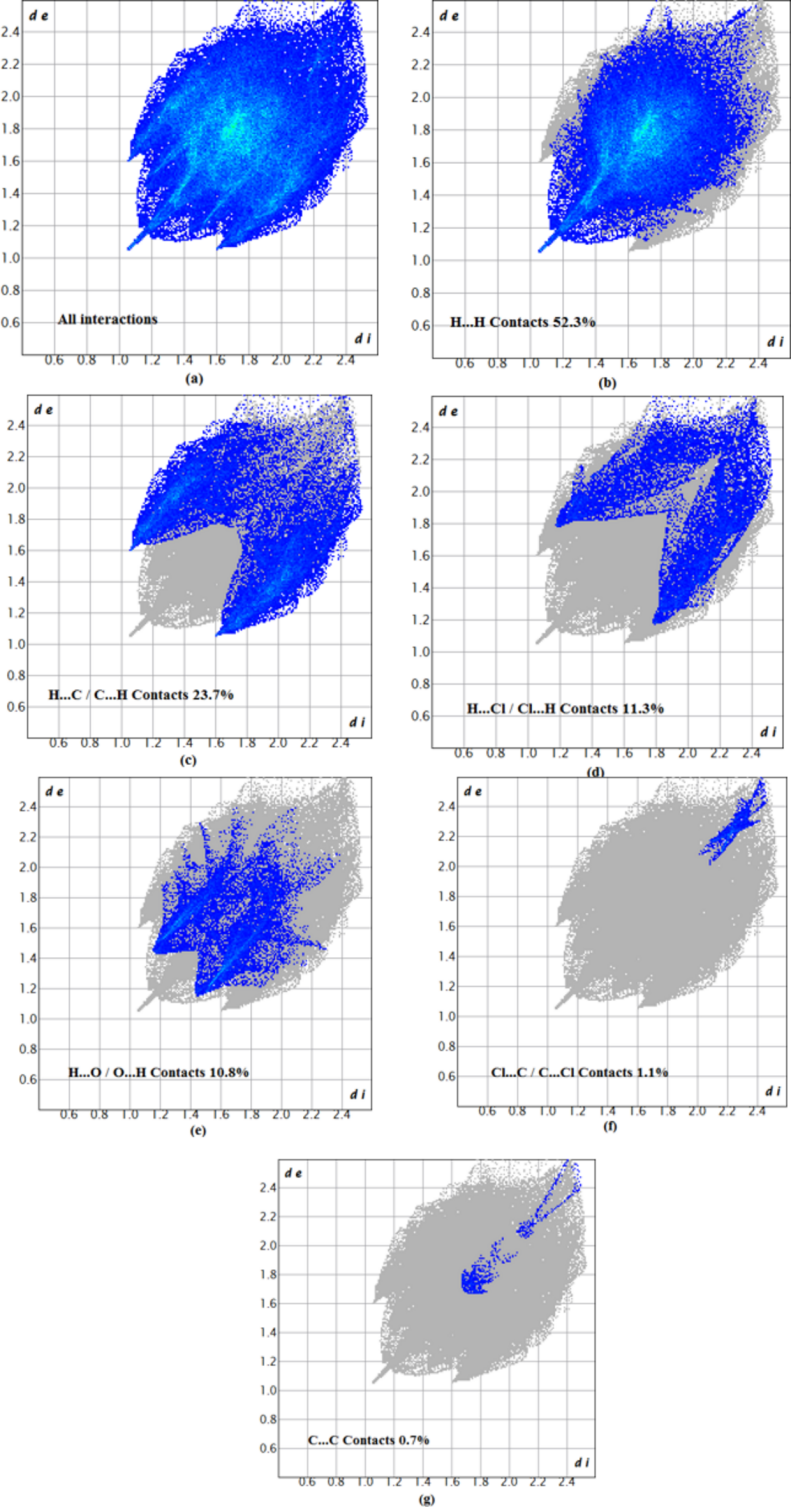
Two-dimensional fingerprint plots of (I), showing (*a*) all inter­actions, and those delineated into (*b*) H⋯H, (*c*) H⋯C/C⋯H, (*d*) H⋯Cl/Cl⋯H, (*e*) H⋯O/O⋯H (*f*) Cl⋯C/C⋯Cl and (*g*) C⋯C inter­actions. The *d*_i_ and *d*_e_ values are the closest inter­nal and external distances (in Å) from given points on the Hirshfeld surface.

The two-dimensional fingerprint plots provide qu­anti­tative information about the non-covalent inter­actions and the crystal packing in terms of the percentage contribution of the inter­atomic contacts (Spackman & McKinnon, 2002 ▸; Ashfaq *et al.*, 2021 ▸). The overall two-dimensional fingerprint plot, Fig. 4 ▸*a*, and those delineated into H⋯H inter­actions (52.3%), H⋯C/C⋯H (23.7%), H⋯Cl/Cl⋯H (11.3%), H⋯O/O⋯H (10.8%), Cl⋯C/C⋯Cl (1.1%) and C⋯C (0.7%) inter­actions are illustrated in Fig. 4 ▸*b*–*g*, respectively. The most important inter­action is H⋯H, which is reflected in Fig. 4 ▸*b* as widely scattered points of high density due to the large hydrogen content of the mol­ecule with the tip at *d*_e_ = *d*_i_ = 1.10 Å. The large number of H⋯H, H⋯C/C⋯H, H⋯Cl/Cl⋯H, H⋯O/O⋯H and Cl⋯C/C⋯Cl inter­actions suggest that van der Waals and hydrogen-bonding inter­actions play the major roles in the crystal packing (Hathwar *et al.*, 2015 ▸). The fragment patches on the HS provide an easy way to investigate the nearest neighbour coordination environment of a mol­ecule, which is 15 in the present case.

## 5. DFT Studies

**Figure 5:**
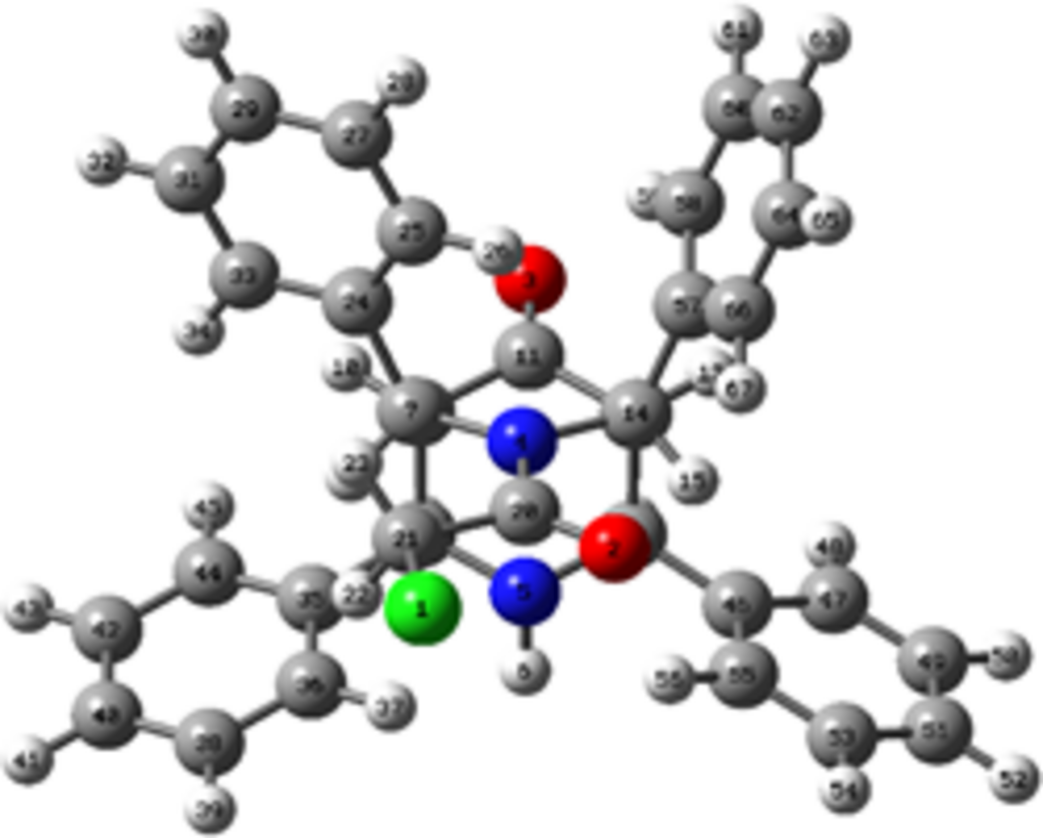
The DFT-optimized structure of (I).

The optimized structure of (I) in the gas phase was computed with *Gaussian 09W* (Frisch *et al.*, 2009 ▸) using the B3LYP/6–31G (d, p) basis set and generated by *GaussView 5.0*. Comparison of the experimentally determined structure parameters by single-crystal X-ray diffraction with that of theoretical ones obtained from the optimized structure revealed that they are in good agreement (Table 2 ▸). The optimized structure of (I) is shown in Fig. 5 ▸.

**Figure 6:**
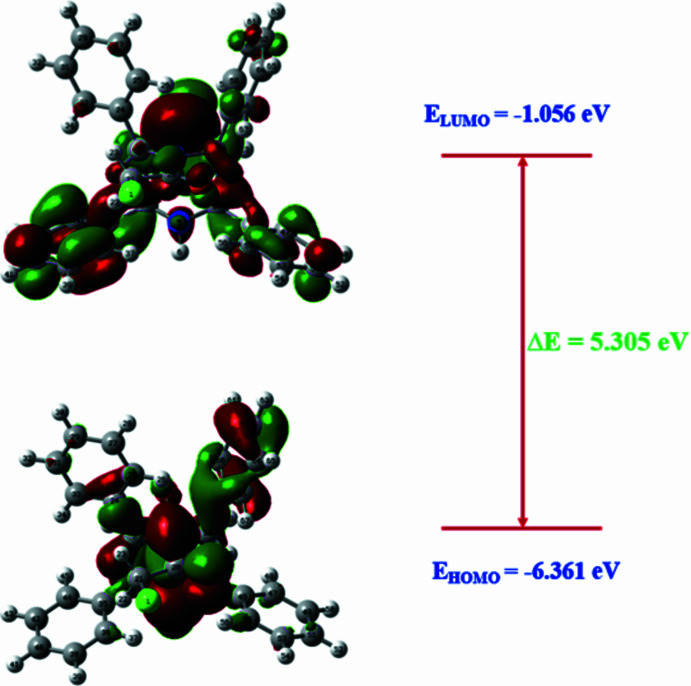
The HOMO/LUMO energy diagram of (I).

The HOMO and LUMO (Fig. 6 ▸) were generated and their energies were evaluated from the optimized structure. The electron density in the HOMO mainly resides on the amidic carbonyl (N—C=O) group and the bicyclic ring system and at the phenyl groups to a lesser extent. In the LUMO, the electronic charge densities are delocalized to reside on the bicyclic ring and the phenyl groups. The energies of HOMO and LUMO are −6.361 eV and −0.1.056 eV, respectively, resulting in an energy gap (Δ*E*) of 5.305 eV.

**Figure 7:**
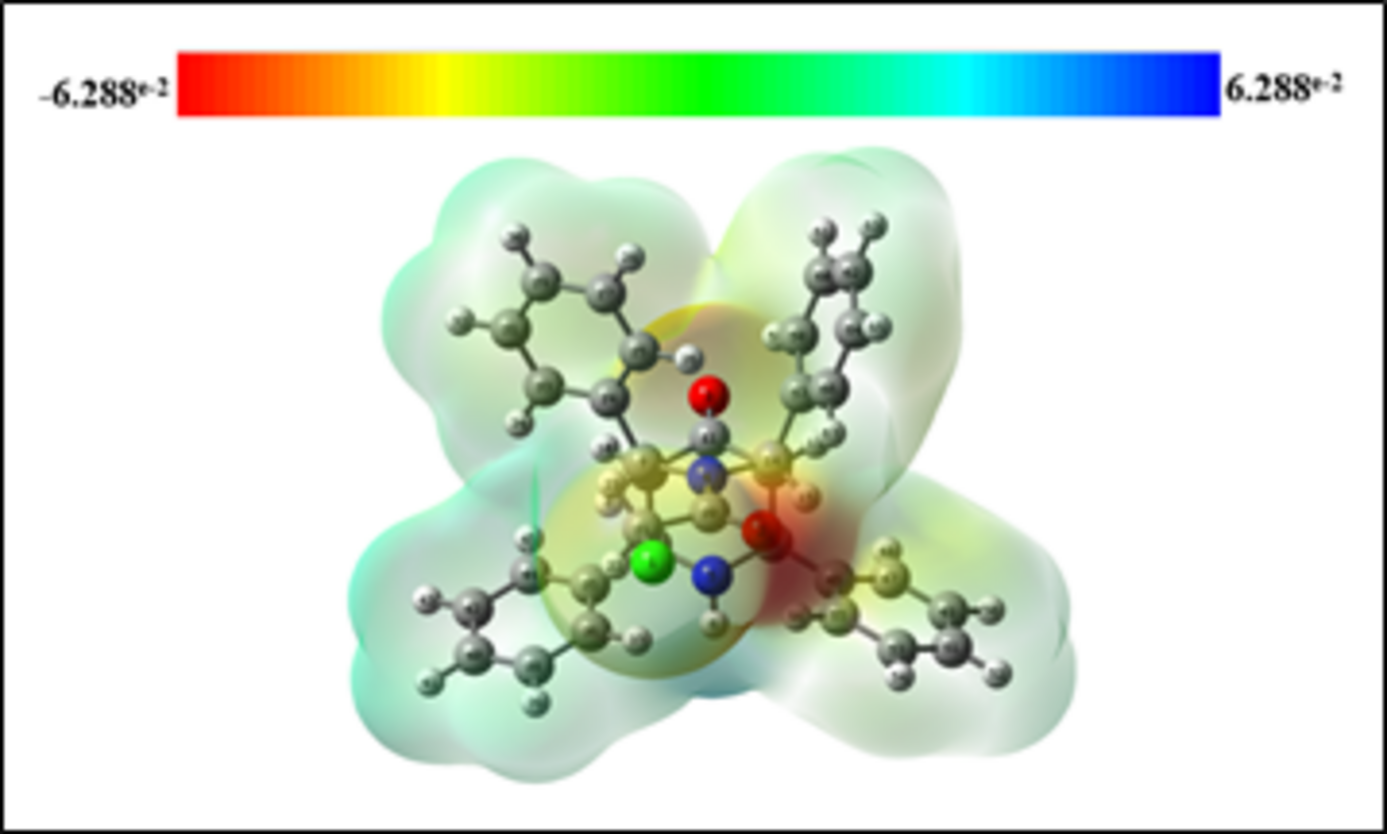
The mol­ecular electrostatic potential surface of (I).

The mol­ecular electrostatic potential surface (MEPS; Fig. 7 ▸) is used to find the positive and negative electrostatic potential of the mol­ecule, which provides possible information about the reactive sites of (I). The electron-rich part with a partial negative charge is shown by red regions on the MEPS over the carbonyl oxygen atom of the chloro­acetyl moiety, which is expected to undergo electrophilic attack. The pale-yellow colour spread over the chlorine atom and the secondary amine (–NH) shows lower electron density regions. The faint blue colour spread all over the mol­ecule implies less electron-deficient parts. The absence of a bright-blue region on the MEPS reveals that there are no possible sites on the mol­ecule for nucleophile attack.

## 6. Mol­ecular Docking Studies

**Figure 8:**
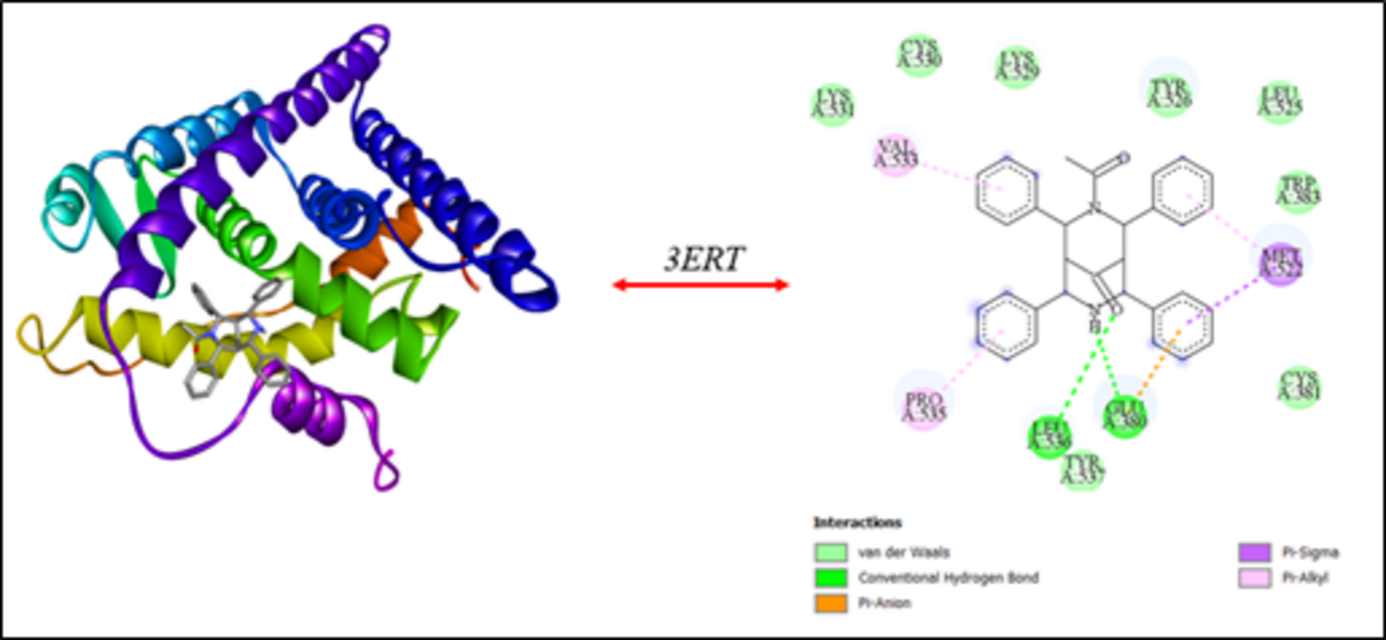
Mol­ecular docking analysis of (I) against 3ERT.

Mol­ecules with ester and acetyl moieties are expected to have enhanced bioavailability and biological activity. Hence, it is inter­esting to evaluate the biological activity of (I) through mol­ecular docking studies. To examine the binding affinity of the title compound, a mol­ecular docking study was performed with ERα protein (PDB ID: 3ERT). The mol­ecular docking was carried out using the *AutoDock* tool (Huey *et al.*, 2012 ▸) and the results were visualized using *Discovery Studio­Visualizer* software (v21.1.0.20298: Biovia, 2017 ▸). The results showed a good binding affinity to the target receptor 3ERT protein with a docking score of −9.56 kcal mol^−1^. The three- and two-dimensional views of the docking inter­actions are shown in Fig. 8 ▸.

## 7. Synthesis and crystallization

Formation of the parent compound 2,4,6,8-tetra­phenyl-3,7-di­aza­bicyclo­[3.3.1]nonan-9-one was achieved by double Mannich reaction of acetone, benzaldehyde and ammonium acetate in the molar ratio of 1:4:2. The obtained product was utilized for the synthesis of compound (I) by reaction of 2,4,6,8-tetra­phenyl-3,7-di­aza­bicyclo­[3.3.1]nonan-9-one with chloro­acetyl chloride in di­chloro­methane using triethyl amine as a catalyst: yield 90%; white solid; IR (ATR, cm^−1^): 2656, 2799 (aromatic C—H stretching), 1718 (C=O stretching), 1654 (amidic carbon­yl). The solid product was collected, washed and recrystallized from methanol to obtain the pure product.

## 8. Refinement

Crystal data, data collection and structure refinement details are summarized in Table 3 ▸. Atom H2 was located from a difference-Fourier map and other H atoms were placed in idealized positions and allowed to ride on their parent atoms with C—H = 0.93–0.98 Å and *U*_iso_(H) = 1.2*U*_eq_ of the parent atom. Reflections 134, 043, 102 and 210 were obstructed from the beam stop and thus were omitted from the refinement.

## Supplementary Material

Crystal structure: contains datablock(s) I, global. DOI: 10.1107/S2056989024008302/wm5730sup1.cif

Structure factors: contains datablock(s) I. DOI: 10.1107/S2056989024008302/wm5730Isup2.hkl

Supporting information file. DOI: 10.1107/S2056989024008302/wm5730Isup3.cml

CCDC reference: 2189597

Additional supporting information:  crystallographic information; 3D view; checkCIF report

## Figures and Tables

**Table 1:** Hydrogen-bond geometry (Å, °) *Cg*1 and *Cg*2 are the centroids of the C28–C33 and C16–C21 rings, respectively.

*D*—H⋯*A*	*D*—H	H⋯*A*	*D*⋯*A*	*D*—H⋯*A*
C5—H5⋯O1	0.98	2.30	2.725 (2)	105
C17—H17⋯N2	0.93	2.46	2.809 (3)	103
C33—H33⋯O1	0.93	2.50	3.214 (3)	134
C11—H11⋯*Cg*1	0.93	2.72	3.625 (3)	166
C24—H24⋯*Cg*2^i^	0.93	2.87	3.628 (3)	140

Symmetry code: (i) 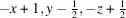.

**Table 2:** Comparison (X-ray and DFT) of selected bond lengths, bond angles and torsion angles (Å, °)

Parameter	SC-XRD	B3LYP/6–31G(d,p)
N1—C8	1.367 (2)	1.367
N1—C1	1.481 (2)	1.4813
N1—C5	1.494 (2)	1.4939
O1=C8	1.224 (2)	1.224
C8—C9	1.528 (2)	1.528
C9—Cl1	1.766 (2)	1.765
N2—C7	1.463 (2)	1.463
N2—C6	1.465 (2)	1.465
C1—N1—C5	122.4 (1)	122.49
C1—N1—C8	120.2 (1)	120.14
C5—N1—C8	116.9 (2)	116.91
N1—C8—C9	116.0 (2)	116.00
N1—C1—C10	116.0 (2)	115.98
N1—C5—C28	111.2 (2)	111.18
N1—C8=O1	122.9 (2)	122.84
C16—C6—C2	110.5 (2)	111.60
C22—C7—N2	111.1 (2)	111.06
N1—C1—C2—C3	–42.8 (2)	–42.77
N1—C5—C4—C3	45.4 (2)	45.38
C3—C2—C1—C10	89.0 (2)	89.05
C10—C1—N1—C5	–97.1 (2)	–97.09
C1—N1—C5—C28	93.9 (2)	93.91
C5—N1—C8=O1	1.4 (3)	1.35
C1—N1—C8=O1	173.9 (2)	173.89
C3—C4—C5—C28	–81.5 (2)	–81.52
C6—N2—C7—C22	–174.7 (2)	–174.68
C7—N2—C6—C16	–179.3 (2)	–179.26

**Table 3:** Experimental details

Crystal data	
Chemical formula	C_33_H_29_ClN_2_O_2_
*M*_r_	521.03
Crystal system, space group	Orthorhombic, *Pbca*
Temperature (K)	298
*a*, *b*, *c* (Å)	9.229 (3), 19.235 (6), 30.058 (10)
*V* (Å^3^)	5336 (3)
*Z*	8
Radiation type	Mo *K*α
μ (mm^−1^)	0.18
Crystal size (mm)	0.35 × 0.23 × 0.19

Data collection	
Diffractometer	Bruker D8 Quest XRD
Absorption correction	Multi-scan (*SADABS*; Krause *et al.*, 2015 ▸)
*T*_min_, *T*_max_	0.692, 0.746
No. of measured, independent and observed [*I* > 2σ(*I*)] reflections	127372, 7493, 4101
*R*_int_	0.104
(sin θ/λ)_max_ (Å^−1^)	0.719

Refinement	
*R*[*F*^2^ > 2σ(*F*^2^)], *wR*(*F*^2^), *S*	0.070, 0.151, 1.07
No. of reflections	7493
No. of parameters	344
No. of restraints	1
H-atom treatment	H-atom parameters constrained
Δρ_max_, Δρ_min_ (e Å^−3^)	0.24, −0.28

Computer programs: *APEX3* and *SAINT* (Bruker, 2017 ▸), *SHELXT* (Sheldrick, 2015*a* ▸), *SHELXL* (Sheldrick, 2015*b* ▸), *ORTEP-3 for Windows* (Farrugia, 2012 ▸), *PLATON* (Spek, 2020 ▸) and *publCIF* (Westrip, 2010 ▸).

## supplementary crystallographic information

### 3-(2-Chloroacetyl)-2,4,6,8-tetraphenyl-3,7-diazabicyclo[3.3.1]nonan-9-one Crystal data

**Table:**

C_33_H_29_ClN_2_O_2_	*D*_x_ = 1.297 Mg m^−^^3^
*M**_r_* = 521.03	Mo *K*α radiation, λ = 0.71073 Å
Orthorhombic, *P**b**c**a*	Cell parameters from 9200 reflections
*a* = 9.229 (3) Å	θ = 2.5–29.5°
*b* = 19.235 (6) Å	µ = 0.18 mm^−^^1^
*c* = 30.058 (10) Å	*T* = 298 K
*V* = 5336 (3) Å^3^	Block, colorless
*Z* = 8	0.35 × 0.23 × 0.19 mm
*F*(000) = 2192	

### 3-(2-Chloroacetyl)-2,4,6,8-tetraphenyl-3,7-diazabicyclo[3.3.1]nonan-9-one Data collection

**Table:**

Bruker D8 Quest XRD diffractometer	4101 reflections with *I* > 2σ(*I*)
Detector resolution: 7.3910 pixels mm^-1^	*R*_int_ = 0.104
ω and Phi Scans scans	θ_max_ = 30.8°, θ_min_ = 2.1°
Absorption correction: multi-scan (*SADABS*; Krause *et al.*, 2015)	*h* = −12→12
*T*_min_ = 0.692, *T*_max_ = 0.746	*k* = −25→25
127372 measured reflections	*l* = −40→42
7493 independent reflections	

### 3-(2-Chloroacetyl)-2,4,6,8-tetraphenyl-3,7-diazabicyclo[3.3.1]nonan-9-one Refinement

**Table:**

Refinement on *F*^2^	Hydrogen site location: mixed
Least-squares matrix: full	H-atom parameters constrained
*R*[*F*^2^ > 2σ(*F*^2^)] = 0.070	*w* = 1/[σ^2^(*F*_o_^2^) + (0.0448*P*)^2^ + 2.2149*P*] where *P* = (*F*_o_^2^ + 2*F*_c_^2^)/3
*wR*(*F*^2^) = 0.151	(Δ/σ)_max_ < 0.001
*S* = 1.07	Δρ_max_ = 0.24 e Å^−^^3^
7493 reflections	Δρ_min_ = −0.28 e Å^−^^3^
344 parameters	Extinction correction: SHELXL (Sheldrick, 2015b), Fc^*^=kFc[1+0.001xFc^2^λ^3^/sin(2θ)]^-1/4^
1 restraint	Extinction coefficient: 0.0032 (4)

### 3-(2-Chloroacetyl)-2,4,6,8-tetraphenyl-3,7-diazabicyclo[3.3.1]nonan-9-one Special details

**Table:**

Geometry. All esds (except the esd in the dihedral angle between two l.s. planes) are estimated using the full covariance matrix. The cell esds are taken into account individually in the estimation of esds in distances, angles and torsion angles; correlations between esds in cell parameters are only used when they are defined by crystal symmetry. An approximate (isotropic) treatment of cell esds is used for estimating esds involving l.s. planes.

### 3-(2-Chloroacetyl)-2,4,6,8-tetraphenyl-3,7-diazabicyclo[3.3.1]nonan-9-one Fractional atomic coordinates and isotropic or equivalent isotropic displacement parameters (Å^2^)

**Table:**

	*x*	*y*	*z*	*U*_iso_*/*U*_eq_	
Cl1	0.49791 (8)	0.40099 (3)	0.00094 (2)	0.0784 (2)	
O1	0.35619 (16)	0.31422 (7)	0.06683 (5)	0.0595 (4)	
O2	−0.01614 (17)	0.45163 (9)	0.20859 (5)	0.0695 (5)	
N1	0.23075 (16)	0.39715 (7)	0.10473 (5)	0.0386 (3)	
N2	0.38457 (18)	0.40602 (8)	0.19171 (6)	0.0496 (4)	
H2	0.459922	0.401953	0.208188	0.060*	
C1	0.2156 (2)	0.47202 (9)	0.11529 (6)	0.0409 (4)	
H1	0.310034	0.492740	0.108463	0.049*	
C2	0.1919 (2)	0.48463 (10)	0.16565 (6)	0.0436 (4)	
H2A	0.153292	0.531690	0.169613	0.052*	
C3	0.0874 (2)	0.43415 (11)	0.18594 (6)	0.0475 (5)	
C4	0.1368 (2)	0.36052 (10)	0.18000 (6)	0.0459 (5)	
H4	0.064183	0.329585	0.193177	0.055*	
C5	0.1534 (2)	0.34183 (9)	0.13028 (6)	0.0417 (4)	
H5	0.214691	0.300281	0.129005	0.050*	
C6	0.3352 (2)	0.47838 (10)	0.19303 (6)	0.0474 (5)	
H6	0.314926	0.490966	0.224002	0.057*	
C7	0.2784 (2)	0.35584 (10)	0.20830 (6)	0.0492 (5)	
H7	0.253838	0.369213	0.238841	0.059*	
C8	0.3309 (2)	0.37562 (10)	0.07418 (6)	0.0431 (4)	
C9	0.4103 (2)	0.43311 (11)	0.04895 (7)	0.0508 (5)	
H9A	0.481483	0.454309	0.068419	0.061*	
H9B	0.341384	0.468752	0.040346	0.061*	
C10	0.1061 (2)	0.51234 (10)	0.08728 (6)	0.0464 (5)	
C11	−0.0107 (2)	0.48393 (12)	0.06587 (7)	0.0584 (6)	
H11	−0.025562	0.436142	0.066996	0.070*	
C12	−0.1073 (3)	0.52561 (15)	0.04244 (9)	0.0750 (7)	
H12	−0.186720	0.505394	0.028441	0.090*	
C13	−0.0874 (3)	0.59549 (15)	0.03974 (8)	0.0791 (8)	
H13	−0.152993	0.622987	0.024194	0.095*	
C14	0.0291 (4)	0.62480 (14)	0.05989 (10)	0.0940 (10)	
H14	0.044147	0.672500	0.057854	0.113*	
C15	0.1261 (3)	0.58363 (12)	0.08362 (9)	0.0777 (8)	
H15	0.205677	0.604246	0.097254	0.093*	
C16	0.4491 (2)	0.52762 (11)	0.17489 (7)	0.0505 (5)	
C17	0.5834 (3)	0.50516 (13)	0.16039 (8)	0.0678 (6)	
H17	0.609601	0.458846	0.164171	0.081*	
C18	0.6794 (3)	0.55085 (17)	0.14029 (10)	0.0867 (8)	
H18	0.770121	0.535105	0.131307	0.104*	
C19	0.6422 (4)	0.61856 (17)	0.13359 (10)	0.0874 (9)	
H19	0.705128	0.648347	0.118731	0.105*	
C20	0.5127 (3)	0.64236 (15)	0.14871 (10)	0.0831 (8)	
H20	0.488173	0.688855	0.144833	0.100*	
C21	0.4167 (3)	0.59769 (11)	0.16994 (8)	0.0649 (6)	
H21	0.329674	0.614934	0.180959	0.078*	
C22	0.3419 (2)	0.28339 (10)	0.20994 (7)	0.0499 (5)	
C23	0.3028 (3)	0.23822 (12)	0.24377 (8)	0.0667 (6)	
H23	0.236247	0.252547	0.265113	0.080*	
C24	0.3613 (4)	0.17210 (13)	0.24627 (10)	0.0826 (8)	
H24	0.333283	0.142401	0.269117	0.099*	
C25	0.4597 (3)	0.15019 (13)	0.21554 (11)	0.0802 (8)	
H25	0.500679	0.106173	0.217809	0.096*	
C26	0.4979 (3)	0.19359 (14)	0.18116 (10)	0.0777 (8)	
H26	0.563727	0.178566	0.159800	0.093*	
C27	0.4384 (3)	0.25990 (13)	0.17818 (8)	0.0653 (6)	
H27	0.463899	0.288685	0.154583	0.078*	
C28	0.0095 (2)	0.32249 (10)	0.10794 (7)	0.0462 (5)	
C29	−0.1247 (2)	0.33945 (12)	0.12559 (8)	0.0619 (6)	
H29	−0.130584	0.361836	0.152957	0.074*	
C30	−0.2510 (3)	0.32299 (14)	0.10237 (10)	0.0774 (7)	
H30	−0.340461	0.335275	0.114241	0.093*	
C31	−0.2453 (3)	0.28918 (14)	0.06257 (10)	0.0811 (8)	
H31	−0.330293	0.278831	0.047312	0.097*	
C32	−0.1135 (3)	0.27048 (13)	0.04513 (9)	0.0743 (7)	
H32	−0.108948	0.246764	0.018200	0.089*	
C33	0.0127 (2)	0.28700 (11)	0.06772 (7)	0.0582 (5)	
H33	0.101471	0.274039	0.055682	0.070*	

### 3-(2-Chloroacetyl)-2,4,6,8-tetraphenyl-3,7-diazabicyclo[3.3.1]nonan-9-one Atomic displacement parameters (Å^2^)

**Table:**

	*U*^11^	*U*^22^	*U*^33^	*U*^12^	*U*^13^	*U*^23^
Cl1	0.0978 (5)	0.0704 (4)	0.0671 (4)	0.0026 (3)	0.0425 (3)	0.0029 (3)
O1	0.0677 (10)	0.0457 (8)	0.0652 (10)	0.0056 (7)	0.0204 (8)	−0.0010 (7)
O2	0.0682 (10)	0.0708 (11)	0.0694 (10)	0.0045 (8)	0.0287 (8)	−0.0056 (8)
N1	0.0407 (8)	0.0373 (8)	0.0378 (8)	−0.0006 (6)	0.0034 (6)	0.0023 (6)
N2	0.0494 (9)	0.0474 (9)	0.0520 (10)	−0.0019 (8)	−0.0099 (8)	0.0070 (8)
C1	0.0453 (10)	0.0391 (10)	0.0383 (9)	−0.0006 (8)	0.0025 (8)	0.0013 (8)
C2	0.0483 (10)	0.0443 (10)	0.0381 (10)	0.0036 (8)	0.0025 (8)	−0.0013 (8)
C3	0.0494 (11)	0.0588 (12)	0.0344 (10)	0.0002 (9)	0.0038 (8)	0.0007 (9)
C4	0.0470 (11)	0.0495 (11)	0.0412 (10)	−0.0025 (9)	0.0057 (8)	0.0080 (8)
C5	0.0432 (10)	0.0382 (9)	0.0435 (10)	−0.0011 (8)	0.0040 (8)	0.0056 (8)
C6	0.0561 (12)	0.0469 (11)	0.0391 (10)	−0.0002 (9)	−0.0024 (9)	−0.0008 (8)
C7	0.0579 (12)	0.0519 (12)	0.0377 (10)	−0.0021 (10)	−0.0001 (9)	0.0071 (9)
C8	0.0431 (10)	0.0447 (11)	0.0416 (10)	0.0006 (8)	0.0025 (8)	0.0015 (8)
C9	0.0542 (12)	0.0510 (12)	0.0471 (11)	−0.0020 (9)	0.0132 (9)	0.0012 (9)
C10	0.0576 (12)	0.0467 (11)	0.0350 (10)	0.0089 (9)	0.0023 (8)	0.0035 (8)
C11	0.0609 (13)	0.0573 (13)	0.0571 (13)	0.0031 (11)	−0.0054 (10)	0.0134 (10)
C12	0.0672 (15)	0.0894 (19)	0.0684 (16)	0.0095 (14)	−0.0133 (12)	0.0213 (14)
C13	0.100 (2)	0.0821 (19)	0.0557 (15)	0.0361 (16)	−0.0093 (14)	0.0132 (13)
C14	0.150 (3)	0.0517 (15)	0.0802 (19)	0.0222 (17)	−0.031 (2)	0.0112 (13)
C15	0.109 (2)	0.0478 (13)	0.0759 (17)	0.0001 (13)	−0.0314 (15)	0.0079 (12)
C16	0.0570 (12)	0.0500 (12)	0.0444 (11)	−0.0061 (10)	−0.0074 (9)	−0.0007 (9)
C17	0.0652 (15)	0.0633 (15)	0.0748 (16)	−0.0032 (12)	0.0063 (12)	0.0003 (12)
C18	0.0678 (17)	0.100 (2)	0.093 (2)	−0.0159 (16)	0.0152 (15)	0.0021 (17)
C19	0.087 (2)	0.092 (2)	0.0830 (19)	−0.0347 (18)	−0.0039 (16)	0.0204 (16)
C20	0.090 (2)	0.0612 (16)	0.098 (2)	−0.0219 (15)	−0.0215 (17)	0.0189 (14)
C21	0.0655 (14)	0.0529 (13)	0.0764 (16)	−0.0059 (11)	−0.0082 (12)	0.0005 (11)
C22	0.0531 (11)	0.0499 (11)	0.0466 (11)	−0.0044 (9)	−0.0101 (9)	0.0094 (9)
C23	0.0793 (16)	0.0631 (14)	0.0577 (13)	−0.0072 (12)	−0.0052 (12)	0.0171 (11)
C24	0.104 (2)	0.0573 (15)	0.0869 (19)	−0.0117 (15)	−0.0241 (17)	0.0251 (14)
C25	0.0850 (19)	0.0498 (14)	0.106 (2)	0.0005 (13)	−0.0447 (17)	0.0077 (15)
C26	0.0627 (15)	0.0737 (17)	0.097 (2)	0.0162 (13)	−0.0125 (14)	−0.0071 (15)
C27	0.0622 (14)	0.0659 (15)	0.0679 (15)	0.0082 (12)	0.0007 (12)	0.0135 (12)
C28	0.0469 (11)	0.0395 (10)	0.0522 (11)	−0.0045 (8)	−0.0010 (9)	0.0093 (9)
C29	0.0493 (12)	0.0663 (14)	0.0701 (15)	−0.0063 (11)	0.0045 (11)	0.0013 (11)
C30	0.0467 (13)	0.0821 (18)	0.104 (2)	−0.0105 (12)	−0.0020 (13)	0.0098 (16)
C31	0.0672 (17)	0.0705 (17)	0.105 (2)	−0.0197 (14)	−0.0286 (16)	0.0136 (16)
C32	0.0855 (19)	0.0634 (15)	0.0741 (16)	−0.0155 (13)	−0.0214 (14)	−0.0014 (12)
C33	0.0613 (13)	0.0535 (12)	0.0597 (13)	−0.0067 (10)	−0.0044 (10)	−0.0018 (10)

### 3-(2-Chloroacetyl)-2,4,6,8-tetraphenyl-3,7-diazabicyclo[3.3.1]nonan-9-one Geometric parameters (Å, º)

**Table:**

Cl1—C9	1.766 (2)	C14—C15	1.392 (4)
O1—C8	1.224 (2)	C14—H14	0.9300
O2—C3	1.220 (2)	C15—H15	0.9300
N1—C8	1.367 (2)	C16—C17	1.383 (3)
N1—C1	1.481 (2)	C16—C21	1.389 (3)
N1—C5	1.494 (2)	C17—C18	1.387 (3)
N2—C6	1.465 (2)	C17—H17	0.9300
N2—C7	1.463 (2)	C18—C19	1.362 (4)
N2—H2	0.8574	C18—H18	0.9300
C1—C10	1.527 (3)	C19—C20	1.358 (4)
C1—C2	1.549 (3)	C19—H19	0.9300
C1—H1	0.9800	C20—C21	1.389 (4)
C2—C3	1.499 (3)	C20—H20	0.9300
C2—C6	1.562 (3)	C21—H21	0.9300
C2—H2A	0.9800	C22—C27	1.382 (3)
C3—C4	1.499 (3)	C22—C23	1.385 (3)
C4—C5	1.545 (3)	C23—C24	1.384 (3)
C4—C7	1.562 (3)	C23—H23	0.9300
C4—H4	0.9800	C24—C25	1.362 (4)
C5—C28	1.534 (3)	C24—H24	0.9300
C5—H5	0.9800	C25—C26	1.375 (4)
C6—C16	1.516 (3)	C25—H25	0.9300
C6—H6	0.9800	C26—C27	1.391 (3)
C7—C22	1.512 (3)	C26—H26	0.9300
C7—H7	0.9800	C27—H27	0.9300
C8—C9	1.528 (3)	C28—C29	1.387 (3)
C9—H9A	0.9700	C28—C33	1.389 (3)
C9—H9B	0.9700	C29—C30	1.395 (3)
C10—C11	1.369 (3)	C29—H29	0.9300
C10—C15	1.388 (3)	C30—C31	1.363 (4)
C11—C12	1.391 (3)	C30—H30	0.9300
C11—H11	0.9300	C31—C32	1.373 (4)
C12—C13	1.359 (4)	C31—H31	0.9300
C12—H12	0.9300	C32—C33	1.385 (3)
C13—C14	1.356 (4)	C32—H32	0.9300
C13—H13	0.9300	C33—H33	0.9300
			
C8—N1—C1	120.15 (14)	C14—C13—C12	119.5 (2)
C8—N1—C5	116.92 (15)	C14—C13—H13	120.3
C1—N1—C5	122.48 (14)	C12—C13—H13	120.3
C6—N2—C7	114.17 (16)	C13—C14—C15	120.1 (3)
C6—N2—H2	108.9	C13—C14—H14	119.9
C7—N2—H2	106.6	C15—C14—H14	119.9
N1—C1—C10	115.97 (15)	C10—C15—C14	121.1 (3)
N1—C1—C2	112.04 (14)	C10—C15—H15	119.4
C10—C1—C2	111.46 (15)	C14—C15—H15	119.4
N1—C1—H1	105.5	C17—C16—C21	117.5 (2)
C10—C1—H1	105.5	C17—C16—C6	122.6 (2)
C2—C1—H1	105.5	C21—C16—C6	119.7 (2)
C3—C2—C1	112.78 (15)	C18—C17—C16	120.8 (2)
C3—C2—C6	106.31 (15)	C18—C17—H17	119.6
C1—C2—C6	112.53 (15)	C16—C17—H17	119.6
C3—C2—H2A	108.4	C19—C18—C17	120.6 (3)
C1—C2—H2A	108.4	C19—C18—H18	119.7
C6—C2—H2A	108.4	C17—C18—H18	119.7
O2—C3—C2	123.54 (19)	C20—C19—C18	119.7 (3)
O2—C3—C4	124.42 (18)	C20—C19—H19	120.2
C2—C3—C4	111.57 (16)	C18—C19—H19	120.2
C3—C4—C5	111.42 (15)	C19—C20—C21	120.4 (3)
C3—C4—C7	104.16 (16)	C19—C20—H20	119.8
C5—C4—C7	115.51 (16)	C21—C20—H20	119.8
C3—C4—H4	108.5	C20—C21—C16	120.8 (2)
C5—C4—H4	108.5	C20—C21—H21	119.6
C7—C4—H4	108.5	C16—C21—H21	119.6
N1—C5—C28	111.18 (15)	C27—C22—C23	118.0 (2)
N1—C5—C4	112.27 (15)	C27—C22—C7	121.91 (18)
C28—C5—C4	113.22 (16)	C23—C22—C7	120.1 (2)
N1—C5—H5	106.5	C24—C23—C22	121.0 (3)
C28—C5—H5	106.5	C24—C23—H23	119.5
C4—C5—H5	106.5	C22—C23—H23	119.5
N2—C6—C16	111.60 (17)	C25—C24—C23	120.5 (3)
N2—C6—C2	108.79 (15)	C25—C24—H24	119.7
C16—C6—C2	110.45 (16)	C23—C24—H24	119.7
N2—C6—H6	108.6	C24—C25—C26	119.5 (3)
C16—C6—H6	108.6	C24—C25—H25	120.2
C2—C6—H6	108.6	C26—C25—H25	120.2
N2—C7—C22	111.08 (17)	C25—C26—C27	120.3 (3)
N2—C7—C4	109.67 (15)	C25—C26—H26	119.9
C22—C7—C4	113.26 (16)	C27—C26—H26	119.9
N2—C7—H7	107.5	C22—C27—C26	120.6 (2)
C22—C7—H7	107.5	C22—C27—H27	119.7
C4—C7—H7	107.5	C26—C27—H27	119.7
O1—C8—N1	122.85 (17)	C29—C28—C33	117.9 (2)
O1—C8—C9	121.15 (17)	C29—C28—C5	123.29 (19)
N1—C8—C9	115.99 (16)	C33—C28—C5	118.83 (18)
C8—C9—Cl1	111.84 (14)	C28—C29—C30	120.1 (2)
C8—C9—H9A	109.2	C28—C29—H29	119.9
Cl1—C9—H9A	109.2	C30—C29—H29	119.9
C8—C9—H9B	109.2	C31—C30—C29	121.0 (3)
Cl1—C9—H9B	109.2	C31—C30—H30	119.5
H9A—C9—H9B	107.9	C29—C30—H30	119.5
C11—C10—C15	117.5 (2)	C30—C31—C32	119.6 (2)
C11—C10—C1	125.28 (18)	C30—C31—H31	120.2
C15—C10—C1	117.21 (19)	C32—C31—H31	120.2
C10—C11—C12	120.9 (2)	C31—C32—C33	119.8 (3)
C10—C11—H11	119.6	C31—C32—H32	120.1
C12—C11—H11	119.6	C33—C32—H32	120.1
C13—C12—C11	120.9 (3)	C32—C33—C28	121.5 (2)
C13—C12—H12	119.6	C32—C33—H33	119.3
C11—C12—H12	119.6	C28—C33—H33	119.3
			
C8—N1—C1—C10	90.8 (2)	C2—C1—C10—C15	74.2 (2)
C5—N1—C1—C10	−97.09 (19)	C15—C10—C11—C12	−1.8 (3)
C8—N1—C1—C2	−139.68 (16)	C1—C10—C11—C12	177.6 (2)
C5—N1—C1—C2	32.4 (2)	C10—C11—C12—C13	0.8 (4)
N1—C1—C2—C3	−42.8 (2)	C11—C12—C13—C14	0.5 (4)
C10—C1—C2—C3	89.03 (19)	C12—C13—C14—C15	−0.8 (5)
N1—C1—C2—C6	77.48 (19)	C11—C10—C15—C14	1.4 (4)
C10—C1—C2—C6	−150.70 (16)	C1—C10—C15—C14	−178.0 (2)
C1—C2—C3—O2	−129.2 (2)	C13—C14—C15—C10	−0.1 (5)
C6—C2—C3—O2	107.1 (2)	N2—C6—C16—C17	−1.7 (3)
C1—C2—C3—C4	58.3 (2)	C2—C6—C16—C17	−122.9 (2)
C6—C2—C3—C4	−65.43 (19)	N2—C6—C16—C21	174.43 (18)
O2—C3—C4—C5	128.2 (2)	C2—C6—C16—C21	53.3 (2)
C2—C3—C4—C5	−59.4 (2)	C21—C16—C17—C18	−2.2 (4)
O2—C3—C4—C7	−106.6 (2)	C6—C16—C17—C18	174.1 (2)
C2—C3—C4—C7	65.80 (18)	C16—C17—C18—C19	−1.4 (4)
C8—N1—C5—C28	−93.73 (19)	C17—C18—C19—C20	3.3 (5)
C1—N1—C5—C28	93.93 (19)	C18—C19—C20—C21	−1.6 (4)
C8—N1—C5—C4	138.26 (16)	C19—C20—C21—C16	−2.0 (4)
C1—N1—C5—C4	−34.1 (2)	C17—C16—C21—C20	3.8 (3)
C3—C4—C5—N1	45.4 (2)	C6—C16—C21—C20	−172.5 (2)
C7—C4—C5—N1	−73.2 (2)	N2—C7—C22—C27	−35.9 (3)
C3—C4—C5—C28	−81.5 (2)	C4—C7—C22—C27	88.0 (2)
C7—C4—C5—C28	159.92 (16)	N2—C7—C22—C23	144.60 (19)
C7—N2—C6—C16	−179.28 (15)	C4—C7—C22—C23	−91.5 (2)
C7—N2—C6—C2	−57.2 (2)	C27—C22—C23—C24	1.5 (3)
C3—C2—C6—N2	57.01 (19)	C7—C22—C23—C24	−179.0 (2)
C1—C2—C6—N2	−66.9 (2)	C22—C23—C24—C25	0.4 (4)
C3—C2—C6—C16	179.81 (16)	C23—C24—C25—C26	−1.7 (4)
C1—C2—C6—C16	55.9 (2)	C24—C25—C26—C27	1.1 (4)
C6—N2—C7—C22	−174.69 (16)	C23—C22—C27—C26	−2.1 (3)
C6—N2—C7—C4	59.3 (2)	C7—C22—C27—C26	178.4 (2)
C3—C4—C7—N2	−59.65 (19)	C25—C26—C27—C22	0.9 (4)
C5—C4—C7—N2	62.9 (2)	N1—C5—C28—C29	−109.3 (2)
C3—C4—C7—C22	175.63 (16)	C4—C5—C28—C29	18.2 (3)
C5—C4—C7—C22	−61.8 (2)	N1—C5—C28—C33	69.8 (2)
C1—N1—C8—O1	173.88 (18)	C4—C5—C28—C33	−162.69 (17)
C5—N1—C8—O1	1.4 (3)	C33—C28—C29—C30	−2.1 (3)
C1—N1—C8—C9	−6.6 (2)	C5—C28—C29—C30	177.0 (2)
C5—N1—C8—C9	−179.11 (16)	C28—C29—C30—C31	1.1 (4)
O1—C8—C9—Cl1	15.7 (3)	C29—C30—C31—C32	0.5 (4)
N1—C8—C9—Cl1	−163.88 (14)	C30—C31—C32—C33	−1.0 (4)
N1—C1—C10—C11	24.6 (3)	C31—C32—C33—C28	−0.1 (4)
C2—C1—C10—C11	−105.2 (2)	C29—C28—C33—C32	1.7 (3)
N1—C1—C10—C15	−156.0 (2)	C5—C28—C33—C32	−177.5 (2)

### 3-(2-Chloroacetyl)-2,4,6,8-tetraphenyl-3,7-diazabicyclo[3.3.1]nonan-9-one Hydrogen-bond geometry (Å, º)

*Cg*1 and *Cg*2 are the centroids of the C28–C33 and C16–C21 rings, respectively.

**Table:**

*D*—H···*A*	*D*—H	H···*A*	*D*···*A*	*D*—H···*A*
C5—H5···O1	0.98	2.30	2.725 (2)	105
C17—H17···N2	0.93	2.46	2.809 (3)	103
C33—H33···O1	0.93	2.50	3.214 (3)	134
C11—H11···*Cg*1	0.93	2.72	3.625 (3)	166
C24—H24···*Cg*2^i^	0.93	2.87	3.628 (3)	140

Symmetry code: (i) −*x*+1, *y*−1/2, −*z*+1/2.
